# Prevalence of multiple organ dysfunction in the pediatric intensive care unit: Pediatric Risk of Mortality III *versus* Pediatric Logistic Organ Dysfunction scores for mortality prediction

**DOI:** 10.5935/0103-507X.20170029

**Published:** 2017

**Authors:** Azza Abd Elkader El Hamshary, Seham Awad El Sherbini, HebatAllah Fadel Elgebaly, Samah Abdelkrim Amin

**Affiliations:** 1 Department of Pediatrics, Faculty of Medicine, Cairo University - Cairo, Egypt.; 2 Department of Pediatric Intensive Care, Faculty of Medicine, Cairo University - Cairo, Egypt.

**Keywords:** Multiple organ failure, Intensive care units, pediatric/statistics & numerical data, Child

## Abstract

**Objectives:**

To assess the frequency of primary multiple organ failure and the role of sepsis as a causative agent in critically ill pediatric patients; and calculate and evaluate the accuracy of the Pediatric Risk of Mortality III (PRISM III) and Pediatric Logistic Organ Dysfunction (PELOD) scores to predict the outcomes of critically ill children.

**Methods:**

Retrospective study, which evaluated data from patients admitted from January to December 2011 in the pediatric intensive care unit of the Children's Hospital of the University of Cairo.

**Results:**

Out of 237 patients in the study, 72% had multiple organ dysfunctions, and 45% had sepsis with multiple organ dysfunctions. The mortality rate in patients with multiple organ dysfunction was 73%. Independent risk factors for death were mechanical ventilation and neurological failure [OR: 36 and 3.3, respectively]. The PRISM III score was more accurate than the PELOD score in predicting death, with a Hosmer-Lemeshow X^2^ (Chi-square value) of 7.3 (df = 8, p = 0.5). The area under the curve was 0.723 for PRISM III and 0.78 for PELOD.

**Conclusion:**

A multiple organ dysfunctions was associated with high mortality. Sepsis was the major cause. Pneumonia, diarrhea and central nervous system infections were the major causes of sepsis. PRISM III had a better calibration than the PELOD for prognosis of the patients, despite the high frequency of the multiple organ dysfunction syndrome.

## Abstract (pt)

**Objetivo:**

Avaliar a frequência de falência de múltiplos órgãos primária e o papel da sepse como agente causal em pacientes pediátricos críticos; e calcular e avaliar a precisão dos escores *Pediatric Risk of Mortality III* (PRISM III) e *Pediatric Logistic Organ Dysfunction* (PELOD) para predizer os desfechos de crianças em estado crítico.

**Métodos:**

Estudo retrospectivo, que avaliou dados de pacientes admitidos entre janeiro a dezembro de 2011 na unidade de terapia intensiva pediátrica do *Children's Hospital da Cairo University*.

**Resultados:**

Dentre os 237 pacientes estudo, 72% tiveram falência de múltiplos órgãos e 45% sepse com falência de múltiplos órgãos. A taxa de mortalidade em pacientes com falência de múltiplos órgãos foi de 73%. Os fatores independentes de risco para óbito foram ventilação mecânica e falência neurológica (OR: 36 e 3,3, respectivamente). O PRISM III foi mais preciso para prever óbito, com qui quadrado no teste de Hosmer-Lemeshow de 7,3 (df = 8; p = 0,5). A área sob a curva foi de 0,723 para o PRISM III e de 0,78 para o PELOD.

**Conclusão:**

A falência de múltiplos órgãos esteve associada à elevada mortalidade. A sepse foi sua principal causa. Pneumonia, diarreia e infecções do sistema nervoso central foram as principais causas de sepse. O PRISM III teve melhor calibração do que o PELOD para prognóstico dos pacientes, apesar da elevada frequência da síndrome de falência de múltiplos órgãos.

## INTRODUCTION

The World Health Organization (WHO) estimates that 10 million children die annually worldwide and that 99% of these deaths occur in developing countries. Acute respiratory disease and malaria are the most common causes of death in children under five years of age in developing countries.^(1)^ Many of these children are at risk for multiple organ dysfunction syndrome (MODS), which is a major cause of death in the intensive care unit (ICU).^(2)^ A dysregulated immune response or immune paralysis in which homeostasis between the pro-inflammatory and anti-inflammatory reactions is lost is thought to be key in the development of MODS.^(3)^ For instance, the IL-8 levels are clearly associated with worsened organ dysfunction within 24 hours.^(4)^

Various scoring systems to predict ICU morbidity and mortality have been developed over the last 30 years. According to Gregoire and Russel, these scoring systems serve four major purposes: they help identify suitable candidates for clinical trials; they quantify the severity of illness for administrative decisions, such as resource allocation; they serve as an audit tool to assess ICU performance and quality of care; and they help predict patient outcomes.^(5)^ A score that predicts the severity of MODS in critically ill children would be a key outcome measure.

Two scoring systems [the Pediatric Logistic Organ Dysfunction (PELOD) and the Pediatric Risk of Mortality (PRISM)] are used to quantify the physiological status and can be used to compute the expected mortality risk. The PELOD score is derived from the MODS criteria. Because the MODS is closely associated with pediatric intensive care unit (PICU) mortality, the PELOD score can be considered a surrogate for the probability of death.^(6)^ The PRISM III score is an updated, second-generation scoring system that includes more than 11,000 consecutive admissions in 32 PICUs and has been validated for use in the United States.^(7)^ PRISM III is a widely accepted standard against which other scores are compared. The Pediatric Index of Mortality (PIM) and its updated version (PIM2) are additional prognostic scores that are used in the medical literature.^(8)^ Evaluation of the performances of PIM and PIM2 in PICUs from low- and middle-income countries have reported excellent "discrimination" but poor "calibration" of the scores.^(9)^ Therefore, the aim of this study is to determine the frequency and outcomes of critically ill children admitted to PICUs with primary MODS. Another aim of this study is to assess the frequency of MODS secondary to sepsis in PICU patients. The study also compares the diagnostic accuracy of the PRISM III and PELOD scores via calibration and discrimination in predictions of the prognosis in these children.

## METHODS

This retrospective study assessed data from 237 patients admitted from January 2011 to December 2011 in the PICU of Children's Hospital, Cairo University. Our goals were to estimate the frequency of day one multiple organ dysfunction and to determine whether the PELOD or PRISM III score calculated on the first day was superior for prognostic predictions. Organ dysfunction was given a numerical value using the PELOD score calculated within the first 24 hours of MODS presentation.^(10)^ The 16 variables of the PRISM III scoring scale were also applied in the first 24 hours of PICU admission.

Sepsis was defined and classified according to the 2005 International Consensus Conference on Pediatric Sepsis.^(11)^ We also used the organ dysfunction criteria adapted by Proulx.^(12)^ The study excluded trauma, burns, and postoperative cardiac surgery cases, which were admitted to other specialized units. The study received Ethics Committee approval.

The receiver operating characteristic curve (ROC) helps evaluate the diagnostic ability of tests by discriminating the true state of subjects, finding the optimal cut-off values, and comparing two alternative diagnostic tasks when both tasks are performed on the same subject.^(13)^ We plotted the sensitivity *versus* 1-specifity via the receiver operating characteristic curve and the area under the curve (AUC). Calibration refers to the agreement between the observed and expected (predicted) outcomes. Models are considered well-calibrated when the expected and observed event rates are similar across different subgroups (deciles) of fitted risk values. The Hosmer-Lemeshow goodness-of-fit analysis was performed to calibrate both scores. A p value > 0.05 indicated acceptable calibration.^(14)^ We applied Cox, log rank, and regression analyses. We also collected data on the age, gender, reason for admission, length of hospital stay, need for ventilation, duration of ventilation, postoperative state, post-cardiopulmonary resuscitation state, sepsis, number of organ system failures, Glasgow coma score, and need for inotropes.

## RESULTS

The study included 237 patients (134 males and 103 females) with a median age of 12 months. Of the 237 patients, 72% had MODS, and 45% had sepsis with MODS. Lung failure secondary to respiratory tract infection was the primary initial diagnosis (32%), followed by postoperative major surgery (16.5%). The study included other etiologies of MODS, including exposure to toxic drugs, intracranial hemorrhage, inborn error of metabolism presenting with acute crises, and graft rejection in colon interposition surgeries. The median length of the PICU stay was seven days (Table 1). We compared patients admitted with single organ dysfunction and with MODS (Table 2). The most prevalent organ dysfunction was acute kidney injury. Of the 94 patients (40%) who died, the mortality rates for single organ dysfunction and MODS were 27% and 73%, respectively. Tables 3 and 4 show the independent predictors for a long hospital stay and mortality in the patients with MODS.

**Table 1:** Descriptive analysis of the study population

Variables N = 237	N (%)
Age	
1 year or less	134 (56.5)
> 1 year	103 (43.5)
Median, range (months)	12 (1 - 144)
Sex	
Male	134 (56.5)
Diagnosis on admission	
Respiratory tract infection	75 (31.6)
Postoperative	39 (16.5)
Gastroenteritis	13 (5.5)
CNS infection	10 (4.2)
Guillain-Barre syndrome	11 (4.6)
Intracranial hemorrhage	8 (3.4)
Status epileptics	8 (3.4)
Inborn error of metabolism	5 (2.1)
Exogenous intoxication	5(2.1)
Aplastic anemia	2 (0.8)
PELOD Score, median (range)	12 (1 - 52)
PRISM III Score, median (range)	19 (9 - 42)
Single organ dysfunction	66 (27.8)
Multiple organ dysfunction	171 (72.2)
Site of sepsis In MODS	
Sepsis with MODS	77/171 (45)
Respiratory	44/77 (57)
Blood stream infection	10/77 (13)
GIT	13/77 (17)
CNS	10/77 (13)
Leukocyte count (x10^3^/L)	12.38 (1.1 - 48)
Leucopenia	19 (8)
Leukocytosis	121(51)
Mechanical ventilation	156 (65.8)
Duration of mechanical ventilation, median (range)	2 (1 - 120) days
Median, (range)	7 (1 - 144) days
> 7 days	115 (48.5)
7 days or less	122 (51.5)
Discharge	143 (60.3)
Overall deaths	94 (39.7)

NS - central nervous system; PELOD - Pediatric Logistic Organ Dysfunction; PRISM - Pediatric Risk of Mortality; MODS - multiple organ dysfunction syndrome; GIT - gastrointestinal tract.

**Table 2:** Comparative analysis of the study population according to either single or multiple organ failure

Variables	SOF (28%) N (%)	MODS (72%) N (%)	p value
Age			
Median (range)	12 m (1 - 144)	12 m (1 - 144)	0.801
Sex			
Male	40/134 (29.9)	94/134 (70.1)	0.433
Need for ventilation	46/156 (29.5)	110/156 (70.5)	0.0211
Duration of MV, median (range)	3 days (1 - 29)	2 days (1 - 120)	0.607
Hospital stay in days			
7 days	33/115 (28.7)	82/115 (71.3)	0.777
Median (min-max)	7.5 (1 - 45)	7 (21-120)	0.636
Mortality	25 (26.6)	69 (73.4)	0.727
Metabolic failure	20/97 (20.6)	77/97 (79.4)	0.039*
Acute kidney injury	3/31 (9.7)	28/31 (90.3)	0.015*
Neurological failure	25/103 (24.3)	78/103 (75.7)	0.282
Cardiovascular failure	34/139 (24.5)	105/139 (75.5)	0.166
Respiratory failure	54/198 (27.3)	144/198 (72.7)	0.656
Hematological failure	17/69 (24.6)	52/69 (75.4)	0.480
Hepatic insult	3/23 (13)	20/23 (87)	0.096
Platelet count (x10^3^/L), median (range)	321 (35 - 994)	330 (13 - 1368)	0.548
Leukocyte count (x10^3^/L), median (range)	(2 - 48)	(0 - 40)	0.206

m - months; SOF - single organ failure; MODS - multiple organ dysfunction syndrome; MV - mechanical ventilation.

* p value below 0.05 is considered significant.

**Table 3:** Multivariate logistic regression analysis identifying independent factors of a prolonged hospital stay in multiple organ dysfunction syndrome patients

Variables	β	OR	95%Cl	p value
Mechanical ventilation	1.568	4.797	1.744 - 13.194	0.002
Acute kidney injury	1.196	3.306	1.086 - 10.065	0.035
Inotrope use	0.989	2.688	1.062 - 6.804	0.037
Cardiovascular failure	0.883	2.419	1.015 - 5.766	0.046
Age ≤ 1 year	0.807	2.242	1.044 - 4.815	0.038

OR - odds ratio; 95%CI - 95% confidence interval.

**Table 4:** Regression analysis for risk factors of death in multiple organ dysfunction syndrome patients

Variables	β	OR	95%Cl	p value
Ventilation	3.473	35.616	3.743 - 42.6	0.002
Neurological failure	1.205	3.338	1.285 - 8.860	0.015

OR - odds ratio; 95%CI - 95% confidence interval.

**Figure 1:**
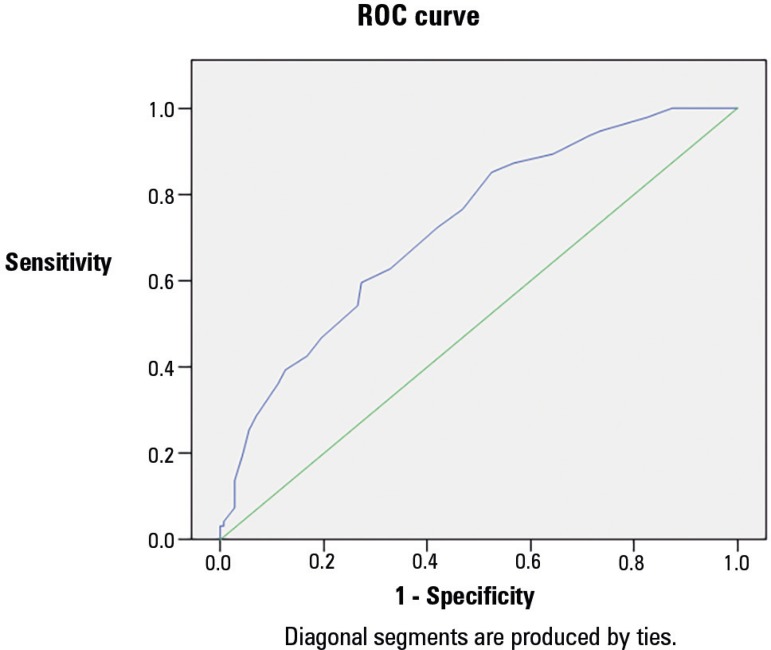
Receiver operating characteristic (ROC) curve analysis of the PRISM III score for the prediction of mortality. ROC - receiver operating characteristic.

**Figure 2:**
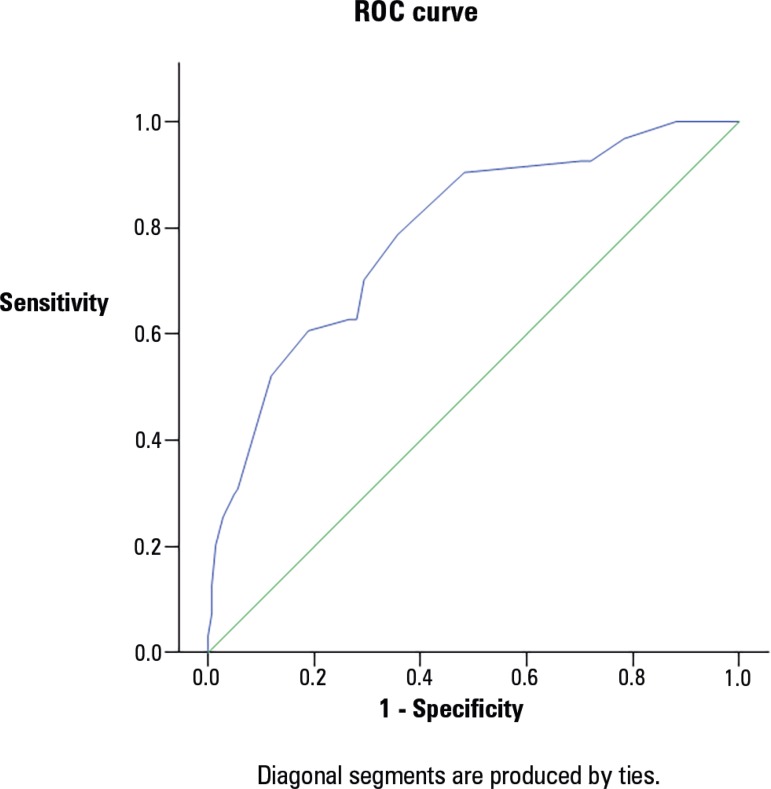
Receiver operating characteristic (ROC) curve analysis of the PELOD score for the prediction of mortality.

The PRISM III score had good calibration, as predicted by estimating the differences between the observed and expected mortalities across all deciles of the mortality risk for the PRISM III scores that were statistically insignificant. As shown in table 5, the Hosmer-Lemeshow X^2^ (Chi-square value) was 7.3 (degree of freedom - df = 8, p = 0.5). The PELOD score had poor calibration using the Hosmer-Lemeshow test. A significant difference was found between the observed and expected mortality rates across the deciles of risk. As shown in table 6, the Hosmer-Lemeshow X^2^ (Chi-square value) was 29.9 (df = 7, p < 0.001). Figure 1 shows the results of the receiver operating characteristic analysis, which indicated that the PRISM III score showed acceptable discrimination (AUC=0.726) for the patients who survived compared to those who died and that the PELOD score showed good discrimination (AUC = 0.788) between death and survival. Figures 1 and 2 and table 7 showed that the survival probability declined for PRISM III scores greater than 20 and for PELOD scores greater than 13 (p ≤ 0.001).

**Table 5:** Hosmer-Lemeshow goodness-of-fit analysis of the Pediatric Logistic Organ Dysfunction score and standardized mortality ratio across all deciles of risk

PELOD score	Survivors (n = 143)		Non-survivors (n = 94)		SMR	95%CI	p value
Deciles of risk	Observed	Expected	Observed	Expected			
1	17	15,002	0	1,998	NA	NA	NA
2	23	25,993	7	4,007	1,747	0.764 - 3.456	0.135
3	34	26,674	2	9,326	0,214	0.036 - 0.709	0.016
4	18	20,630	11	8,370	1,314	0.691 - 2.284	0.363
5	11	17,745	15	8,255	1,817	1.056 - 2.930	0.019
6	23	16,240	10	16,760	0,597	0.303 - 1.064	0.099
7	9	12,970	20	16,030	1,248	0.783 - 1.893	0.321
8	7	6,232	17	17,768	0,957	0.576 - 1.501	0.855
9	1	1,513	12	11,487	1,045	0.566 - 1.776	0.880

PELOD - Pediatric Logistic Organ Dysfunction; SMR - standardized mortality ratio; 95%CI - 95% confidence interval; NA - not applicable. Hosmer-Lemeshow X^2^ (Chi-square value) = 29.9; degree of freedom = 7; p < 0.001 → poor calibration.

**Table 6:** Hosmer-Lemeshow goodness-of-fit analysis for the Pediatric Risk of Mortality III score and standardized mortality ratio across all deciles of risk

PRISM III score	Survivors (n=143)		Non-survivors (n=94)		SMR	95%CI	p value
Deciles of risk	Observed	Expected	Observed	Expected			
1	25	22,984	2	4,016	0,498	0.083 - 1.645	0.314
2	16	16,135	4	3,865	1,035	0.329 - 2.496	0.945
3	21	20,420	6	6,580	0,912	0.370 - 1.897	0.821
4	14	16,807	10	7,193	1,390	0.706 - 2.478	0.295
5	20	21,223	13	11,777	1,104	0.614 - 1.840	0.722
6	9	10,055	8	6,945	1,152	0.535 - 2.187	0.689
7	14	13,215	11	11,785	0,933	0.491 - 1.622	0.819
8	14	11,807	13	15,193	0,856	0.476 - 1.426	0.574
9	6	8,310	19	16,690	1,138	0.706 - 1.745	0.572
10	4	2,044	8	9,956	0,804	0.373 - 1.526	0.535

PRISM - Pediatric Risk of Mortality; SMR - standardized mortality ratio; 95%CI - 95% confidence interval. Hosmer-Lemeshow X^2^ (Chi-square value) = 7.3; degree of freedom = 8; p = 0.5 → good calibration.

**Table 7:** Area under the curve analysis for the Pediatric Risk of Mortality III and Pediatric Logistic Organ Dysfunction mortality predictions

	AUC	95%CI	Cut-off	Sensitivity %	Specificity %	PPV %	NPV %	Accuracy %
PELOD	0.788	0.729 - 0.846	≥ 13	70.2	69.9	60.6	78.1	70.0
Prism III	0.726	0.661 - 0.790	≥ 20	63.8	67.1	56.1	73.8	65.9

AUC - area under the curve; 95%CI - 95% confidence interval; PPV - positive predictive value; NPV - negative predictive value; PELOD - Pediatric Logistic Organ Dysfunction; PRISM - Pediatric Risk of Mortality.

## DISCUSSION

MODS is the main cause of death in PICUs. During the one-year observation period of our study, MODS accounted for 72% of ICU admission diagnoses. Other geographic areas have reported rates ranging from 11% to 81%.^(15-20)^ Late arrival to tertiary care, a shortage of pediatric advanced life support program implementation, and PICU bed availability limit admission to the most critical cases. The high incidence of sepsis is also a cofactor for the percentage of MODS cases, with a rate of 45% observed in the study. This rate of MODS with sepsis is higher than the rate mentioned by Typpo et al..^(16)^ The risk factors for a prolonged hospital stay are consistent with those mentioned in previous studies.^(21-23)^ Studies from New Delhi and the United States reported day-one MODS mortality rates of 50% and 10%, respectively.^(15,16)^ Ventilated patients with MODS are 35 times more likely to die, and a Glasgow coma scale below eight triples the risk of mortality. These data differ from the report of Costa et al., which mentioned the need for vasoactive drugs instead of neurological dysfunction as a mortality predictor.^(24)^ Koury et al. mentioned that septic patients admitted to the ICU with a great number of organ failures had a superior mortality rate.^(25)^

Compared to the 16 variables included the PRISM III, the PELOD score is easier to calculate in a busy PICU. PRISM III had good calibration and discrimination in our study, which was compatible with most reports.^(26-30)^ Validation of the PRISM III score outside of North America has shown mixed results. A study from Pakistan by Qureshi et al. showed good discrimination and calibration of the PRISM III (AUC = 0.78 [0.67 - 0.89]; x2 = 7.49, p = 0.49) in their PICU.^(28)^ Choi et al. from China showed that PRISM III accurately predicted mortality in the PICU (AUC = 0.79 [0.65 - 0.98]; p = 0.395).^(29)^ A study from India by Taori et al. showed good discriminatory performance and calibration of the PRISM score.^(30)^ A study from India by Thukral et al. showed that PRISM III under-predicted mortality in their PICU.^(27)^ The new PRISM IV prediction algorithm includes the same PRISM physiological variable ranges with subcategories for neurological and non-neurological PRISM scores, age, admission source, cardiopulmonary arrest within 24 hours before admission, cancer diagnosis, and low-risk systems with primary dysfunction.^(31)^

Two studies have validated the PELOD score.^(32,33)^ In the developmental study for the PELOD, which included 594 consecutive patients and 51 deaths, the discrimination of the PELOD score was 0.98 ± 0.01 (AUC ROC + standard error). The calibration was good (p value = 0.44, 3 degrees of freedom). In a validation study of 1,806 consecutive patients, the PELOD discrimination was 0.91 ± 0.01 (AUC ROC + standard error). The calibration was good (p value = 0.54, 5 degrees of freedom). Our result agrees with the published data showing good discrimination.^(32,33)^ However, the PELOD scores showed poor calibration in differentiating between survival versus death. In 2013, Leteurtre et al. published PELOD-2, which included mean arterial pressure and lactatemia in the cardiovascular dysfunction and excluded hepatic dysfunction. PELOD-2 showed good discrimination and calibration in assessing the severity of organ dysfunction.^(34)^ Accurate information about predicted mortality improves communication with parents about the possible prognosis.

## CONCLUSION

This study investigated the validity of two outcome scoring systems for predictions of outcomes in children with MODS who presented on day one of pediatric intensive care unit admission and were characterized by a high mortality rate and a long pediatric intensive care unit length of stay. The PELOD score had poor calibration in distinguishing death from survival, which was consistent with most external validation studies, despite the high MODS frequency in our unit. The PRISM III score indicated proper calibration in differentiating death from survival. However, further validation of PRISM IV and PELOD 2 is needed in pediatric intensive care unit settings.
